# Post-GWAS methodologies for localisation of functional non-coding variants: *ANGPTL3*

**DOI:** 10.1016/j.atherosclerosis.2015.12.009

**Published:** 2016-03

**Authors:** Federico Oldoni, Jutta Palmen, Claudia Giambartolomei, Philip Howard, Fotios Drenos, Vincent Plagnol, Steve E. Humphries, Philippa J. Talmud, Andrew J.P. Smith

**Affiliations:** a Department of Cardiovascular Genetics, Institute of Cardiovascular Sciences, University College London, London, UK; b Department of Molecular Genetics, University Medical Center Groningen, University of Groningen, Groningen, The Netherlands; c UCL Genetics Institute, University College London, London, UK

**Keywords:** *ANGPTL3*, Polymorphism, Regulation, Chromatin, Genome-wide, Functional polymorphism, FAIRE, LDL-C, GWAS, genome-wide association study, LDL-C, low density lipoprotein cholesterol, HDL-C, high density lipoprotein cholesterol, TG, triglycerides, EMSA, electrophoretic mobility shift assay, SNP, single nucleotide polymorphism

## Abstract

Genome-wide association studies have confirmed the involvement of non-coding angiopoietin-like 3 (*ANGPTL3*) gene variants with coronary artery disease, levels of low-density lipoprotein cholesterol (LDL-C), triglycerides and *ANGPTL3* mRNA transcript. Extensive linkage disequilibrium at the locus, however, has hindered efforts to identify the potential functional variants. Using regulatory annotations from ENCODE, combined with functional *in vivo* assays such as allele-specific formaldehyde-assisted isolation of regulatory elements, statistical approaches including eQTL/lipid colocalisation, and traditional *in vitro* methodologies including electrophoretic mobility shift assay and luciferase reporter assays, variants affecting the *ANGPTL3* regulome were examined. From 253 variants associated with *ANGPTL3* mRNA expression, and/or lipid traits, 46 were located within liver regulatory elements and potentially functional. One variant, rs10889352, demonstrated allele-specific effects on DNA-protein interactions, reporter gene expression and chromatin accessibility, in line with effects on LDL-C levels and expression of *ANGPTL3* mRNA. The *ANGPTL3* gene lies within *DOCK7*, although the variant is within non-coding regions outside of *ANGPTL3*, within *DOCK7*, suggesting complex long-range regulatory effects on gene expression. This study illustrates the power of combining multiple genome-wide datasets with laboratory data to localise functional non-coding variation and provides a model for analysis of regulatory variants from GWAS.

## Highlights

• Over 200 variants exist in strong linkage disequilibrium with the lead *ANGPTL3* SNP locus.
• rs10889352 affects DNA-protein interactions from liver nuclear extract.
• rs10889352 affects reporter gene expression in liver cells.
• rs10889352 is associated with chromatin accessibility.
• Effects on *ANGPTL3* gene expression and lipid levels is likely due to rs10889352.

## 1 Introduction

Genome-wide association studies (GWAS) have revealed novel gene loci and confirmed many candidate genes for numerous traits including lipid levels and coronary artery disease (CAD) risk [1], [2]. Despite the increasing number of loci associated with such traits, identification of functional or causal variants at each locus has not been met with similar advances. This lack of progress is largely due to two important characteristics: the presence of many variants in strong linkage disequilibrium (LD) with the leading GWAS signal, and the fact that the majority of potentially causal SNPs are located in non-coding regions of the genome, for which *in silico* tools to reliably predict SNP functionality are lacking.

A typical example of this occurs at the angiopoietin-like 3 (ANGPTL-3) gene locus, associated with triglyceride (TG) levels, low-density lipoprotein cholesterol (LDL-C) levels, and CAD risk in several independent GWAS [1], [3], [4], where the functional variant(s) associated with these traits have not been identified. ANGPTL-3 is a member of the angiopoietin-like proteins, a family of secreted glycoproteins, expressed in the liver, containing a signal peptide, N-terminal coiled coil domain and a C-terminal fibrinogen-like domain. ANGPTL-3 can inhibit lipoprotein lipase [5] and endothelial lipase [6], and through the fibrinogen-like domain, induce endothelial cell adhesion and migration [7].

In a large meta-analysis comprising >100,000 individuals, rs2131925, the lead SNP at the *ANGPTL3* locus for TG association (4.9 mg/dL decrease) was also associated with LDL-C levels (1.6 mg/dL decrease) [1]. In addition to these associations with common polymorphism, rare missense mutations in *ANGPTL3* have also been identified through exome-sequencing to be associated with familial combined hypolipidemia, confirming this locus as an important mediator in lipid metabolism [8].

**Fig. 1:**
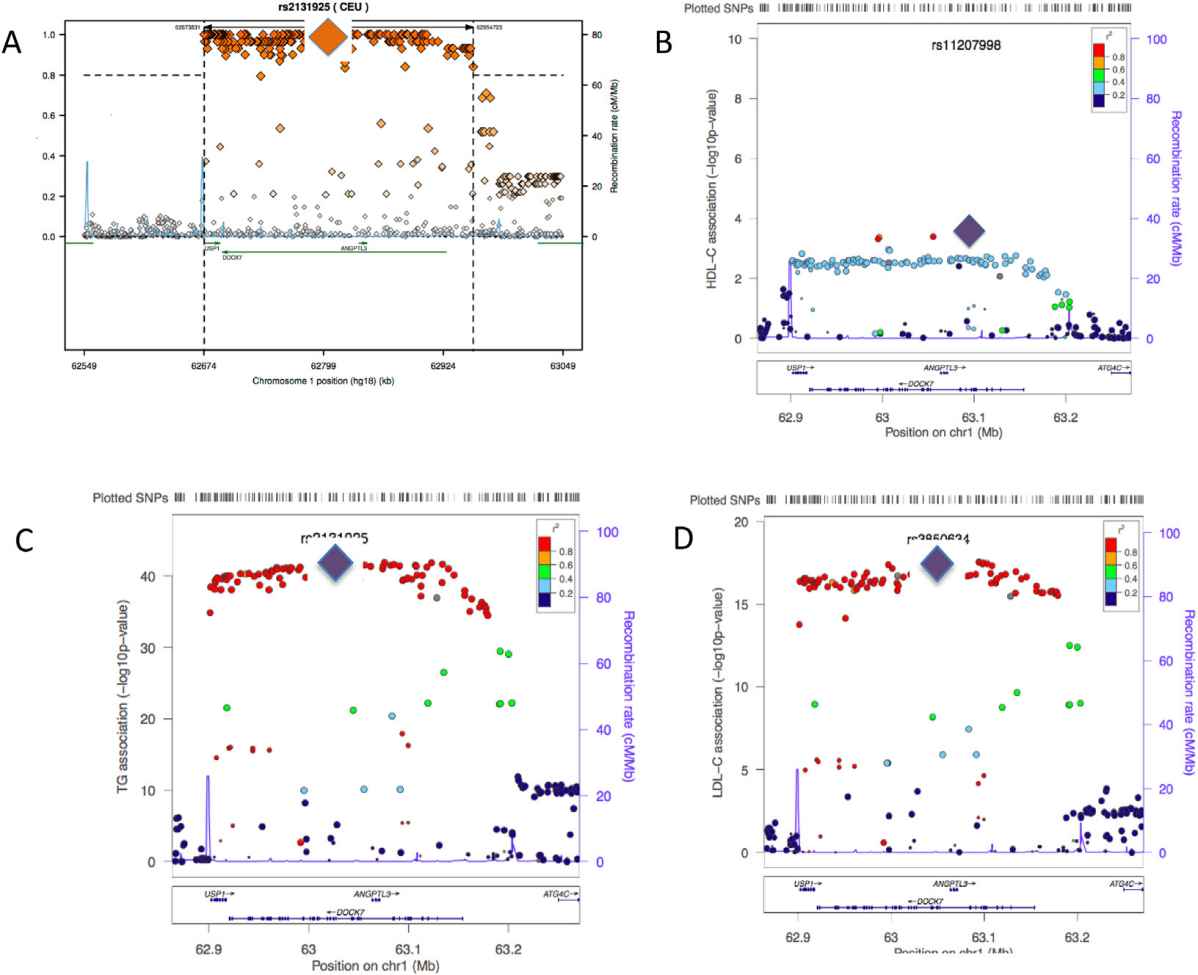
A) Linkage disequilibrium with the lead TG GWAS SNP, rs2131925 (indicated by large diamond), at the *ANGPTL3* locus. Over 200 SNPs are in strong LD at this locus (r^2^ > 0.8), which span 3 genes, *USP1*, *DOCK7* and *ANGPTL3* (http://www.broadinstitute.org/mpg/snap/ldplot.php). 1B) Association plot of HDL-C at the *ANGPTL3* locus, revealing no significant association. 1C) Association plot of TG at *ANGPTL3* locus, showing rs2131925 as lead SNP. 1D) Association plot of LDL-C at *ANGPTL3* locus, showing lead SNP as rs3850634 [1] (http://csg.sph.umich.edu/locuszoom).

*ANGPTL3* lies on the forward strand of chromosome 1p31 within an intronic region of *DOCK7* (located on the reverse strand). Examining the *ANGPTL3* locus, it can be seen that there are over 250 SNPs in very strong LD with the lead TG GWAS SNP that span ∼250 kb and include 3 genes [1], [9], *ANGPTL3*, *DOCK7* and *USP1* (Fig. 1A), leading to the strong likelihood that rs2131925 is simply acting as a marker for the true functional variant(s). Interestingly, SNPs at this locus show a lack of significant association with HDL-C (Fig. 1B), but association with LDL-C shows a similar pattern as for TG, suggesting a common functional polymorphism (Fig. 1C and D).

One commonly employed method to identify potential causal non-coding variants is through *in silico* tools, including databases used to predict the effect of a SNP on transcription factor binding motifs. Such methods, unfortunately, result in many false-positive findings that are not borne out when tested in the laboratory [10]. Functional examination of all SNPs showing association to a trait can be undertaken in the laboratory where the number of SNPs is small [11], [12], although when strong LD within the association signal results in hundreds of potentially causal variants, such analysis is not feasible. The ENCODE and Roadmap Epigenomics projects, however, provide additional tools that can be used to identify non-coding regions of the genome that are likely to harbour important regulatory features [13], [14]. Where such features also contain a genetic variant associated with an epidemiologic trait, this may suggest potentially causality of the variant, rather than simply a marker for the trait.

The lead SNP, rs2131925 is unlikely to represent the functional SNP at the locus, with 92 SNPs in perfect LD, and 202 SNPs in very strong LD (r^2^ > 0.8) [9]. We used data from the ENCODE Project and Roadmap Epigenomics, in addition to epidemiological, statistical and functional assays, to assist in the identification of potentially causal variants at the *ANGPTL3* locus, and examined these variants for functionality in the laboratory.

## 2 Materials and methods

### 2.1 Identification of SNPs associated with lipid biomarkers

Variants in strong LD (r^2^ ≥ 0.8) with the GWAS lead SNP (rs2131925) for TG and LDL-C [14] were identified using 1000 Genomes Project CEU Caucasian population Phase 1 data (1KGP) [9]. Additionally, all variants within 500 kb from *ANGPTL3* reaching genome-wide significance (p < 5 × 10^8^) for LDL-C or TG levels using a large-scale meta-analysis of >100,000 individuals were identified [1] and selected for further analysis.

### 2.2 Identification of variants associated with *ANGPTL3* mRNA transcript levels

Gene expression and genotype data were obtained for 966 post-mortem and surgical resection human liver samples from unrelated European-American individuals [15]. Genotyping was carried out using the Illumina 650Y BeadChip array, and Agilent human gene expression arrays used to profile 39,000 expression probes. Imputation of genotypes not covered on the array was carried out as previously described, and the full methods for this, and the procedures for colocalisation have been detailed previously [16].

### 2.3 Identification of candidate functional SNPs using ENCODE

Published ENCODE annotations for the HepG2 liver cell line for DNaseI-seq, FAIRE-seq, ChIP-seq (transcription factors and histone methylation signatures H3K4me1 and H3K27Ac) were examined in relation to variants associated with lipid biomarkers and *ANGPTL3* transcript levels. Roadmap Epigenomics annotations for liver tissue were examined for DNaseI-seq and histone modifications. SNPs were identified which occurred within narrow peaks for DNaseI hypersensitivity or within 500 bp of SPP narrow peaks for ChIP-seq as previously described [17]. SNPs which were located close to regulatory signatures (<500 bp were also included).

### 2.4 Tissue culture

HepG2 cells were cultured in high-glucose Dulbecco's modified Eagle's medium (PAA, Austria) supplemented with 2 mM l-glutamine and 10% foetal bovine serum (FBS; PAA). EBV-transformed lymphoblast cell lines from the Centre d'Etude du Polymorphism Humain (CEPH) panel (Coriell Cell Repositories), were cultured in RPMI 1640 (PAA) with 2 mM l-glutamine and 15% foetal bovine serum (PAA) at 37 °C, 5% CO_2_. Cell viability was verified using the ADAM-MC cell counter (Digital Bio), and minimum cell viability for experiments was ≥99%. Cytokine stimulation was performed by overnight incubation in serum-free media, followed by addition of 5 ng/ml IL-1β, two hours prior to cell fixing.

### 2.5 *In vitro* analysis of DNA-protein interactions

Nuclear extracts were obtained from HepG2 cells using the NE-PER Nuclear and Cytoplasmic Extraction Reagents Kit (Pierce, USA) as described in the manual, with the addition of Complete Protease Inhibitor (Roche, UK) to buffers CER I and NER I. EMSA probes were designed using 15 bp of genomic sequence on each side of the candidate SNP, and reverse complement for each forward strand was synthesised and annealed (sequences for all probes available on request). Forward sequence for rs6690733: 5′-ATCAAAAGGGGGTAA(**A/C**)CACTACTACGTATAC and rs10889352: 5′-GCAAACAGAAAAAAA(**T/C**)ATAGTACTAGTAAAA-3’.

Probes were labelled using the Biotin 3′-End DNA Labelling Kit (Pierce, USA) as described in the manual, and complementary labelled oligonucleotides annealed by heating to 95 °C for 10 min, followed by slow cooling (−1 °C/min to 30 °C). Each binding reaction consisted of 2 μl 10× binding buffer (100 mM Tris, 500 mM KCl; pH 7.5), 1 μg p[dI-dC], 2 nmol biotin-labelled DNA, made to a total of 20 μl with H_2_O, and incubated at 25 °C for 30 min, followed by the addition of 5× loading buffer. Samples were electrophoresed on a 6% polyacrylamide gel for 150 min at 120 V. Transfer to nylon membrane was achieved through Southern transfer and detected using the Chemiluminescent Nucleic Acid Detection Module (Pierce, USA).

### 2.6 *In silico* analysis of transcription factor binding

To determine which proteins may be interacting with EMSA oligonucleotide probes, the MatInspector (Genomatix, Germany) [18] database was examined using the Transcription Factor Binding Sites (Weight matrices) library for general core promoter elements and vertebrates matrix group. Core similarity (the proportion of bases exactly matching the highest conserved positions of the matrix) was set to 0.75. HaploReg v3 was used as an addition source for predicted SNP effects on transcription factor binding [17].

### 2.7 *In vitro* analysis of gene expression

For the putative *ANGPTL3* regulatory elements surrounding the SNPs of interest, oligonucleotides were designed with the addition of BamHI and SalI restriction sites at the 5’- and 3’-ends and PCR amplification of homozygous wild-type individuals performed. Primer sequences for cloning: rs6690733 - 5′-GGGGCTACTCTTCAAATTCCCT-3′ forward, 5′-GGCCAGCTTCTCTCCCCTAT-3′ reverse; rs10889352 – 5′-TGACACAGGCTAGGTGACAAA-3′ forward, 5′-AGAAGCAGATGCCACCAAGG-3′ reverse. PCR products were digested with BamHI and SalI, and ligated into the BamHI/SalI-linearized pGL3 Promoter vector (Promega), downstream of the SV40 late poly(A) signal. Allelic variants were created using the Site-Directed-Mutagenesis kit (Invitrogen), the variant verified by sequencing and insert digested and re-inserted into a fresh pGL3 vector. The size of insert for rs6690733 was 249 bp and 192 bp for rs10889352.

HepG2 cells were seeded at 2.0 × 10^4^/well in a 96 well plate and grown to confluence in high-glucose Dulbecco's modified Eagle's medium (PAA, Austria) supplemented with 2 mM l-glutamine and 10% fetal bovine serum (FBS). Cells were transfected with 250 ng pGL3 reporter construct with 10 ng pRLTK as a transfection control. Transfection was carried out using Opti-Mem serum-free media (Sigma, Dorset, UK) and Lipofectamine 2000 (Invitrogen, Paisley, UK) as described in the manual. Media was replaced 24 h following transfection, with serum-containing media, and the cells left for 2 days before harvesting. Cells were lysed using Passive Lysis Buffer (Promega, Southampton, UK) and luciferase expression was determined using the Dual Luciferase Reporter Assay System (Promega, Southampton, UK), and measured in the Tropix TR717 Microplate Luminometer (PE Applied Biosystems, UK). Luciferase activity was determined as the mean of 12 transfections with the assay performed in triplicate.

### 2.8 Allele-specific FAIRE

The methodology for allele-specific FAIRE used for this study is described elsewhere [19]. Briefly, 20 unrelated lymphoblast cell lines of European origin (Coriell Repositories) were fixed in the log-phase of growth, and the chromatin extracted. Following sonication, one portion of chromatin was reverse-crosslinked and purified, the other underwent FAIRE enrichment using phenol:chloroform:isoamyl alcohol. Following reversal of crosslinks and DNA purification, all samples were genotyped using the Illumina Metabochip array. Identification of heterozygous individuals at each allele was determined using the B allele frequency (BAF) from the samples not enriched by FAIRE, and the BAF for these individuals compared to that from the FAIRE-treated samples. For rs6690733, which was not present on the Metabochip array, a custom KASPar (KBioscience) genotyping assay was created to determine the allelic imbalance in heterozygotes from extrapolation from a standard curve, and this data was analysed in the same manner as with the Metabochip-derived genotype data (KASPar probes available on request).

### 2.9 Statistical analysis

Luciferase expression was normalised to the pRLTK control vector, and a 2-sided t-test performed between each run of 12 samples. Assays were performed in triplicate. Analysis of FAIRE-gen was performed using a paired t-test between the BAF from SNPs of all heterozygous individuals, comparing non-FAIRE vs FAIRE-treated chromatin. Imputation of genotypes not covered on the array was carried out as described [16] using EUR reference haplotypes available from the 1000 Genomes Project. eQTL analysis was run by fitting a linear regression between expression of the gene and all SNPs between 200 kilobases upstream and downstream from the probe. Colocalisation analysis between eQTL and lipid biomarkers uses summary statistics downloaded from a publicly available meta-analysis (http://www.sph.umich.edu/csg/abecasis/public/lipids2010/) and the statistical methodology is described in full in [16]. A posterior probability >75% was considered as strong evidence for colocalisation.

## 3 Results

### 3.1 Selection of SNPs associated with lipid biomarkers

To identify the pool of variants most likely to contain the functional variant/s responsible for TG and LDL-C plasma levels at the *ANGPTL3* locus, all variants from the 1000 Genomes Project [9] in strong LD (r^2^ > 0.8) with the GWAS lead SNPs (rs2131925 and rs3850634, respectively) were identified, providing 202 SNPs (Supplementary Table 1). Additionally, meta-analysis data from >100,000 individuals with lipid measurements was examined [1], and association at the *ANGPTL3* locus with TG and LDL-C was determined. From this dataset, 151 SNPs reaching genome-wide significance (p < 5 × 10^−8^) were selected. All 106 LDL-C associated SNPs were present within the subset associated with TG (Supplementary Fig. 1). Since the associated polymorphisms are all non-coding variants and therefore likely to be affecting gene expression levels, liver eQTLs for *ANGPTL3* were examined from 966 liver samples, including imputed SNPs from 1KGP not directly genotyped. This data corroborated the association of epidemiological data for lipid levels with *ANGPTL3* transcript levels (Supplementary Figs. 2 and 3). In total, 253 SNPs were identified to show either genome-wide association to a lipid trait or a liver eQTL for *ANGPTL3* within the 0.8 r^2^ cut-off with the TG lead SNP from GWAS, rs2131925 (Supplementary Table 1). Interestingly, it was observed that *DOCK7* transcript levels are also affected by the same variants that affect *ANGPTL3* expression.

### 3.2 Colocalisation of lipid associations and liver eQTLs at the *ANGPTL3* locus

To determine if the association with TG and LDL-C levels at the *ANGPTL3* locus shared a causal variant/s with liver *ANGPTL3* mRNA expression, a Bayesian test to examine colocalisation between pairs of traits was performed (procedure detailed in [16]). Summary statistics from associations with TG and LDL-C were derived from the lipid meta-analysis data used to select *ANGPTL3* variants [1], and the 966-sample liver expression dataset described previously was used for eQTL analysis, with imputation used to detect variants not directly genotyped. The data demonstrated that both TG (posterior probability 92.4%) and LDL-C (posterior probability 90.6%) colocalise with *ANGPTL3* expression, indicating a shared causal variant or variants (Supplementary Fig. 2). Due to the very strong LD at the region, however, colocalisation did not reduce the pool of potential causal variants, and all 253 SNPs were included for further examination to assess functional potential.

### 3.3 Functional assessment of potential regulatory *ANGPTL3* SNPs

Since variants at the *ANGPTL3* locus that determine TG and LDL-C levels colocalise with eQTL markers for *ANGPTL3* transcript levels, a common variant (or variants) affecting gene regulatory processes is likely to be responsible for both associations. The potential for this selection of 253 variants to affect gene regulation was assessed by examining data from the ENCODE Project and Roadmap Epigenomics ^14 13^. Since none of these variants occur within *ANGPTL3* coding regions, 5′ or 3’ UTR, or predicted splice sites, allele-specific regulation is likely to be through effects on promoter, enhancer or silencer elements, rather than effects on mRNA stability. Since the variants are also associated with *DOCK7* transcript levels, a common enhancer/silencer element is more likely to be affected than an *ANGPTL3* promoter element. To identify variants that may affect such regulatory elements, ENCODE and Roadmap Epigenomics datasets were searched for universal signatures of regulatory potential, including DNaseI hypersensitivity (DHS) peaks, histone modifications and FAIRE-seq peaks, and also for transcription factor binding sites. Variants present within, or close (500 bp) to these signatures were also selected for further analysis. 33 SNPs were present directly within an annotated regulatory region in liver cells, and 13 were present close to a regulatory annotation and were included for further analysis due to possible longer-range regulatory roles (Table 1).

### 3.4 Allele-specific protein-binding effects of SNPs within regulatory regions

**Fig. 2:**
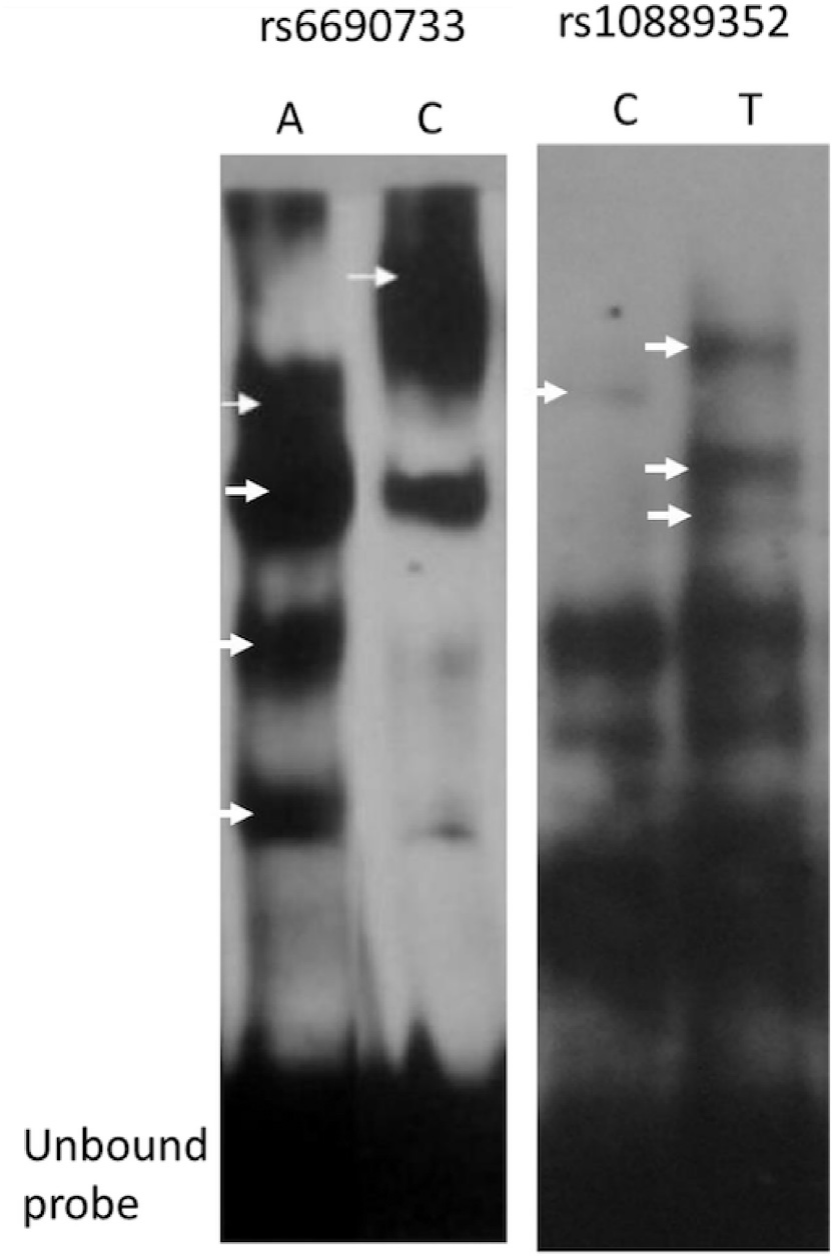
Allele-specific effects on DNA-protein interactions. A HepG2 liver nuclear extract was mixed with allele-specific sequences for all potential regulatory variants from ENCODE analysis and allele-specific FAIRE signals. Binding differences were observed from two variants: rs6690733 and from rs10889352. Arrows indicate where a binding difference was identified between alleles, suggesting a potential effect on gene regulation.

For a non-coding functional variant to affect gene expression, it would be expected to alter binding affinities of transcription factor/s. To assess the potential for each of the 46 lipid-associated variants from ENCODE/Roadmap Epigenomics-derived regulatory regions to affect the interaction of DNA-binding proteins, electrophoretic mobility shift assays (EMSAs) were performed for each of the alleles, using biotin-labelled double-stranded oligonucleotide probes comprising 30bp genomic sequence surrounding each variant, with the variant located directly at the centre of each probe. Each oligonucleotide probe was incubated with a HepG2 liver nuclear extract, and the resulting complexes run on a polyacrylamide gel. From a pool of 46 SNPs, 44 either did not result in transcription factor binding from either allele, or both alleles resulted in identical binding of equal intensity, with the binding site likely to be located outside of the polymorphic site. Two variants demonstrated replicated allele-specific binding to the liver nuclear extract, rs6690733 and rs10889352, indicating changes in transcription factor binding potential, and a possible regulatory role (Fig. 2). Interestingly both variants appear to show multiple proteins bound to the oligonucleotide probes, suggesting a complex means of allele-specific regulation. Replication with a Huh7 nuclear extract demonstrated identical binding patterns to HepG2 nuclear extract (data not shown) indicating a factor common to hepatic cells.

Examination of predicted changes to transcription factor binding based on each allele was examined using two related tools: MatInspector [18] and HaploReg v2 ^17^, which use extensive libraries of matrix descriptions for transcription factor binding sites to identify matches in DNA sequences. Both variants are predicted to alter binding sites for a number of transcription factors (Table 2). Although the two algorithms produced differing results, certain similarities were evident, such as a forkhead box protein with rs10889352 C, and promyelocytic leukaemia zinc finger binding with rs10889352 T. Examining MatInspector or HaploReg v2 results in isolation, there was a similarity in the number of predicted binding factors for each allele as seen in EMSA analysis. To determine if the *in silico* predicted binding was borne out *in vivo*, competitor assays were performed using a panel of unlabelled consensus sequences for 80 transcription factors, including 70 well-characterised transcription factors, and those predicted to be altered *in silico* [20]. This analysis, however, failed to verify the binding of predicted transcription factors or identify additional factors (data not shown), indicating a lack of reliability from prediction algorithms, or tissue-specific effects.

### 3.5 *In vitro* analysis of gene expression

The two SNPs associated with TG levels, *ANGPTL3* transcription and allele-specific DNA-protein interactions are strong candidates for functionality. To examine the effects of these two polymorphisms on gene expression, luciferase reporter assays were performed by designing reporter constructs containing the genomic region surrounding each SNP (200–250 bp) downstream of *luc* reporter gene (acting as a putative enhancer), driving the SV40 promoter in the pGL3 promoter vector. Constructs were transiently transfected into the HepG2 cell line and luciferase activity measured.

**Fig. 3:**
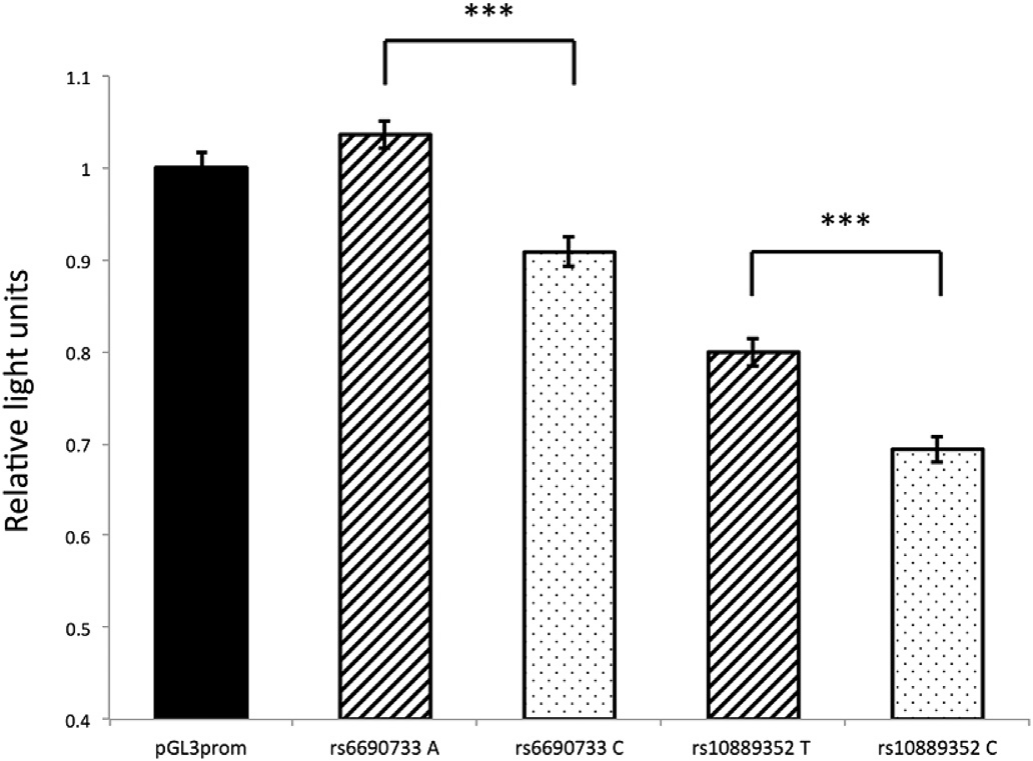
Representative luciferase reporter assays for the two SNPs showing relative allele-specific effects on DNA-protein interactions compared to pGL3 promoter vector. Diagonal hatched columns represent the alleles associated with increased gene expression. Error bars represent standard error, *** = p < 0.001. Assay was repeated three times with 12 wells containing each vector per assay.

Both rs6690733 and rs10889352 showed allele-specific effects on reporter gene expression, with each demonstrating modest reductions (∼10% reduction from each variant; p < 1 × 10^10^) in gene expression for the minor alleles (Fig. 3). Interestingly, both polymorphisms appear to act as silencers in this gene expression model.

### 3.6 *In vivo* analysis of allele-specific effects on chromatin structure

Variants that affect gene transcription which are not located either within a transcription factor protein-coding sequence or regulatory RNA-coding sequence (e.g. miRNA), as is the case at the *ANGPTL3* locus, are likely to be functioning through alteration of DNA-protein interactions at promoter, enhancer or silencer elements. These interactions can either result in, or be altered by, disruption to the local chromatin structure [19]. To assess whether the two potentially functional variants at the *ANGPTL3* locus may have an effect on chromatin structure and to substantiate *in vitro* functionality *in vivo*, allele-specific formaldehyde-assisted-isolation-of-regulatory-elements (FAIRE) was performed. The allelic imbalance was assessed from heterozygotes following FAIRE-enrichment for open chromatin, compared to non-enriched chromatin, using lymphoblast chromatin samples from 20 unrelated individuals.

Interestingly, rs10889352 T > C did indeed demonstrate a robust allele-specific effect on chromatin accessibility (20.4% enrichment of T allele from 10 FAIRE-treated heterozygotes compared to T allele-enrichment from non-treated cells, *p* = 0.002), suggesting that the T allele allows for an increased regulatory potential than the C allele. This data fits with the increased binding of nuclear proteins to the T allele, and higher expression levels in reporter assay, along with the T allele associated with higher *ANGPTL3* transcript levels. The other potentially functional variant at the locus, rs6690733, however did not demonstrate effects on chromatin accessibility (p = 0.53), and is therefore a less plausible casual candidate SNP.

Since expression of the ANGPTL-family respond to inflammatory stimuli [21], allele-specific FAIRE was repeated with lymphoblast cells treated with the inflammatory cytokine IL-1β prior to the fixing of cells for FAIRE, with the hypothesis that transcription factors from inflammatory pathways may exacerbate allele-specific effects on chromatin accessibility. Upon stimulation, rs10889352 showed a similar effect (25.5% enrichment of T allele following FAIRE) and significance level (p = 0.0019), replicating the original effects, suggesting in this case that inflammation does not interact with the functional variant.

## 4 Discussion

Identification of functional variants from GWAS can be a laborious process with large blocks of LD surrounding the lead SNP often resulting in unfeasible numbers of variants to examine in the laboratory. The sequential methodology described here outlines a series of *in silico* and laboratory-based assays to localise functional loci at non-coding regions of the genome, using the *ANGPTL3* locus and its association with TG and LDL-C as an example (Supplementary Fig. 4). Having defined the set of potentially functional SNPs using both epidemiological data and eQTL data from the relevant tissue (liver), data from the ENCODE Project and Roadmap Epigenomics were examined in liver cells to identify regions of regulatory potential surrounding each variant, reducing the number of potentially functional variants to 46. Three complementary laboratory methods were employed to examine the effect of this reduced set of variants on regulatory potential: EMSA, a relatively inexpensive and quick method to identify the effect of variants on DNA-protein interactions; luciferase reporter assay to examine the role of these variants in gene expression; and FAIRE-gen [19], to confirm an *in vivo* role of these variants on chromatin accessibility and therefore regulatory potential.

Allele-specific effects on DNA-protein interactions were observed only for two SNPs (rs6690733 and rs10889352) from of the 46 variants selected from ENCODE, and effects of this manageable number of variants on gene expression were examined by luciferase reporter assays. Both variants also showed allele-specific effects on gene expression, with each allele that showed increased expression levels also associated with increases in *ANGPTL3* transcript expression. Examining *in vivo* effects on chromatin accessibility, only one of the two variants, rs10889352, demonstrated allele-specific effects with the T allele associated with a 20% increase in chromatin accessibility. The rs10889352 T allele may be associated with chromatin accessibility by altering the stability of the nucleosome complex directly from alterations in the DNA sequence, or, more likely from EMSA evidence, by altering the affinity of transcription factors which would in turn, alter chromatin dynamics.

Despite >200 variants in very strong LD with the lead SNP, only one SNP demonstrated likely functionality based on all bioinformatics, *in vivo* and *in vitro* analyses. The variant rs10889352 is located outside of the coding region for *ANGPTL3*, within an intron of *DOCK7* (Supplementary Fig. 5), and close to accessible chromatin determined by FAIRE-seq in HepG2 cells (Supplementary Fig. 6). Several transcription factor binding motifs are predicted to be altered by this variant: EVI1, FOXD3, GR, PLZF and p300 (Table 2). Higher affinity for these factors are predicted for the T allele, which supports the increased protein-DNA interactions for this allele observed in EMSA, and increased reporter gene expression. We could not, however, confirm the identity of these DNA-binding factors, despite assaying for those predicted to bind. This does not necessarily limit the ability to assign functionality to this variant, but does highlight the limitations of using *in silico* tools to correctly predict transcription factor binding. It does, however, restrict the use of chromatin immunoprecipitation to demonstrate *in vivo* interactions, another technique that would help characterise functional variants and determine the precise mechanism of action.

The study described here utilises multiple lines of evidence to demonstrate SNP functionality. Where it is necessary to isolate individual SNPs for analysis, methods such as EMSA and reporter assays are important, but take no account of chromatin accessibility, requiring such information from sources such as ENCODE and Roadmap Epigenomics. FAIRE-gen largely overcomes this weakness, with long-range LD no longer a limiting factor. A potential limitation with the assay described here is the use of allele-specific FAIRE data from lymphoblasts for examining traits seen in the liver. However, although absolute levels of accessible chromatin are tissue-specific, and largely colocalise with tissue-specific transcription factor binding sites [22], evidence from multiple cell lines indicates a correlation of allele-specific effects on accessible chromatin between cell lines [23], providing validity to such analysis. The use of cell lines rather than primary hepatocytes adds one limitation to *in vitro* analysis, and although we replicated DNA-protein interactions with an additional liver cell line, primary hepatoctyes may provide a different result.

This study demonstrates the complexity of identification of SNP functionality in regions where strong LD is present, and where several variants in near-complete LD may be having additive/opposite effects. Such information will be important when examining loci in populations with different LD structures, where knowledge of the functional variant will be able to improve the risk prediction for individuals in these populations compared using a lead tag SNP derived from another population. The methodology described here provides an outline to localise potentially causal regulatory variants based on the use of complementary tools. Reducing the number of functional variants at GWAS loci will allow for the emerging genome-editing techniques such as transcription activator-like effector nucleases [24] and CRISPR/Cas9 [25] to confirm causality, with the ability to examine effects of single variants in cell-based models on gene expression. As the regulome continues to be examined using increasing numbers of cell lines/tissues, and annotations refined with more sophisticated tools, identification of functional elements will significantly improve our ability to predict likely effects of non-coding genetic variants. We have demonstrated here how such annotations can currently help identify potentially functional variants associated with GWAS traits by examining data from primary hepatocytes and HepG2 cells to define regulatory elements in liver cells. The data from these two cell types are likely to cover the majority of regulatory elements, but with further genome-wide analyses, such as ChIP-seq for transcription factors in primary hepatocytes, additional regulatory regions may be discovered at the *ANGPTL3* locus.

## Funding sources

This work was supported by the British Heart Foundation [PG2008/008 to JP and FD, FS/13/6/29977 to AS, SH is a British Heart Foundation chair] and the European Commission [GA No 278397 to PH].

## Disclosures

None.

## Figures and Tables

**Table 1:** Variants located within ENCODE-derived gene regulatory sites.

SNP	Promoter histone signature	Enhancer histone signature	DNaseI hypersensitivity	Proteins bound
rs10889356	X		X	X
rs9436661	X			
rs1168046		X		
rs10789117		X		
rs10889344		X		
rs11207979		X		
rs1168047		X		
rs12029068	X			
rs638714	X			
rs6690733		X		
rs9436221	X			
rs9436222	X			
rs4587594			X	
rs6678483		X		
rs11207997		X		
rs12090886		X		
rs1168045		X		
rs1627591		X		
rs10889337		X		
rs10493322	X		X	
rs1168114		X	X	X
rs1168113		X	X	X
rs12136083		X		
rs6679002		X		
rs12130333		X		
rs10889377		X		
rs17123817		X		
rs10889378		X		
rs13375691			X	X
rs646179			X	X
rs9436223	X			
rs631106	X			X
rs626787	X			
rs10889347				(X)
rs6587980				(X)
rs1184547				(X)
rs10157265				(X)
rs1168015			(X)	(X)
rs12062275				(X)
rs10889336			(X)	(X)
rs1168042			(X)	(X)
rs12037659				(X)
rs11207976				(X)
rs1184865				(X)
rs11207992				(X)
rs10889352		(X)		

X = SNP located within regulatory site.

(X) = SNP located close to regulatory site.

**Table 2:** Comparison of predicted transcription factor binding to putative functional *ANGPTL3* variants using MatInspector and HaploReg v2 databases.

SNP	Allele	MatInspector predicted transcription factor binding	HaploReg v2 predicted transcription factor binding
rs10889352	C	Forkhead family	Forkhead Box Protein D3
rs10889352	T	NKX-homeodomain factors, promyelocytic leukaemia zinc-finger	Ecotropic viral integration site 1/4, Glucocorticoid receptor, promyelocytic leukaemia zinc-finger, p300
rs6690733	A	Barbiturate-inducible element box from pro + eukaryotic genes, forkhead family, GLI zinc finger family, pleomorphic adenoma gene	
rs6690733	C	Barbiturate-inducible element box from pro + eukaryotic genes, hepatic nuclear factor 1, pleomorphic adenoma gene	forkhead box D3, forkhead box O4
