# Supplementary material for: Post-GWAS methodologies for localisation of functional non-coding variants: ANGPTL3
Source: Atherosclerosis. 2016 Mar;246:193–201. doi: 10.1016/j.atherosclerosis.2015.12.009 (PMC4773290; doi:10.1016/j.atherosclerosis.2015.12.009)
Supplement: Supplementary file 1 [file mmc1.docx]

Supplementary Tables and Figures

Supplementary Table 1

|  | TG | LDL-C | LD | eQTL |
| --- | --- | --- | --- | --- |
| rs1168041 | 8.53E-39 | 1.41E-16 | n/a | 1.51E-13 |
| rs1168103 | 1.13E-37 | 3.2E-16 | n/a | n/a |
| rs4915853 | 3.31E-30 | 2.95E-13 | n/a | 7.02E-12 |
| rs6679002 | 8.76E-30 | 3.69E-13 | n/a | 2.33E-11 |
| rs912540 | 3.28E-27 | 2.23E-10 | n/a | 1.54E-08 |
| rs11208010 | 5.81E-23 | 9.56E-10 | n/a | 4.95E-08 |
| rs12117388 | 6.19E-23 | 1.68E-09 | n/a | 1.21E-07 |
| rs12130333 | 7.31E-23 | 1.17E-09 | n/a | 4.01E-08 |
| rs11208008 | 8.42E-23 | 1.22E-09 | n/a | 3.47E-08 |
| rs12092541 | 2.82E-22 | 1.14E-09 | n/a | 1.62E-08 |
| rs11207993 | 6.1E-22 | 6.46E-09 | n/a | 1.03E-07 |
| rs2031373 | 4.02E-21 | n/a | n/a | 1.06E-09 |
| rs11208011 | 1.31E-12 | n/a | n/a | 0.01112 |
| rs11208012 | 3.62E-12 | n/a | n/a | 0.01112 |
| rs6587988 | 1.76E-11 | n/a | n/a | 0.006045 |
| rs12141183 | 1.82E-11 | n/a | n/a | 0.01112 |
| rs12080049 | 1.91E-11 | n/a | n/a | 0.007106 |
| rs7513999 | 2.15E-11 | n/a | n/a | 0.007106 |
| rs10889377 | 2.2E-11 | n/a | n/a | 0.006658 |
| rs17123817 | 2.38E-11 | n/a | n/a | 0.006658 |
| rs11208014 | 2.51E-11 | n/a | n/a | 0.005149 |
| rs12133507 | 2.73E-11 | n/a | n/a | 0.005149 |
| rs10889381 | 3.01E-11 | n/a | n/a | 0.006025 |
| rs12143139 | 4.59E-11 | n/a | n/a | 0.010515 |
| rs4409690 | 4.83E-11 | n/a | n/a | 0.006025 |
| rs12085400 | 4.91E-11 | n/a | n/a | 0.010972 |
| rs10493328 | 5.55E-11 | n/a | n/a | 0.006025 |
| rs4457613 | 5.85E-11 | n/a | n/a | 0.005427 |
| rs11208030 | 6.55E-11 | n/a | n/a | 0.004997 |
| rs12048208 | 7.09E-11 | n/a | n/a | 0.000109 |
| rs11207998 | 7.63E-11 | n/a | n/a | 0.000107 |
| rs11208033 | 9.15E-11 | n/a | n/a | 0.006025 |
| rs10889379 | 9.88E-11 | n/a | n/a | 0.005034 |
| rs12039115 | 1.03E-10 | n/a | n/a | 0.000118 |
| rs10889378 | 1.06E-10 | n/a | n/a | 0.006658 |
| rs10493329 | 1.09E-10 | n/a | n/a | 0.005033 |
| rs17123830 | 1.21E-10 | n/a | n/a | 0.006658 |
| rs12067646 | 1.28E-10 | n/a | n/a | 0.006523 |
| rs11208027 | 1.41E-10 | n/a | n/a | 0.001251 |
| rs7546879 | 1.55E-10 | n/a | n/a | 0.006754 |
| rs11208034 | 1.74E-10 | n/a | n/a | 0.006025 |
| rs12121207 | 1.89E-10 | n/a | n/a | 0.009385 |
| rs11208029 | 1.99E-10 | n/a | n/a | 0.006101 |
| rs17388170 | 2.3E-10 | n/a | n/a | 0.006238 |
| rs11208031 | 2.31E-10 | n/a | n/a | 0.004997 |
| rs11807368 | 2.44E-10 | n/a | n/a | 0.001872 |
| rs4370785 | 2.53E-10 | n/a | n/a | 0.005427 |
| rs11208024 | 8.08E-10 | n/a | n/a | 0.001439 |
| rs13375691 | 5.7E-09 | n/a | n/a | 0.034327 |
| rs11208032 | 3.58E-08 | n/a | n/a | 0.005856 |
| rs1167996 | n/a | n/a | 1 | 1.68E-15 |
| rs624660 | n/a | n/a | 1 | 1.82E-15 |
| rs12037659 | n/a | n/a | 1 | 2.05E-15 |
| rs1629122 | n/a | n/a | 1 | 2.11E-15 |
| rs10889343 | n/a | n/a | 1 | 2.14E-15 |
| rs10889344 | n/a | n/a | 1 | 2.14E-15 |
| rs116405518 | n/a | n/a | 1 | 2.14E-15 |
| rs2131926 | n/a | n/a | 1 | 2.14E-15 |
| rs2366636 | n/a | n/a | 1 | 2.28E-15 |
| rs1748199 | n/a | n/a | 1 | 2.29E-15 |
| rs6690733 | n/a | n/a | 1 | 2.31E-15 |
| rs10789117 | n/a | n/a | 1 | 2.33E-15 |
| rs598253 | n/a | n/a | 1 | 2.38E-15 |
| rs11207986 | n/a | n/a | 1 | 2.39E-15 |
| rs11207969 | n/a | n/a | 1 | 2.43E-15 |
| rs61471917 | n/a | n/a | 1 | 2.44E-15 |
| rs11207979 | n/a | n/a | 1 | 2.49E-15 |
| rs10157265 | n/a | n/a | 1 | 2.5E-15 |
| rs1168047 | n/a | n/a | 1 | 2.51E-15 |
| rs1168050 | n/a | n/a | 1 | 2.52E-15 |
| rs10889339 | n/a | n/a | 1 | 2.58E-15 |
| rs11207983 | n/a | n/a | 1 | 2.58E-15 |
| rs11207984 | n/a | n/a | 1 | 2.58E-15 |
| rs1168020 | n/a | n/a | 1 | 2.58E-15 |
| rs1168021 | n/a | n/a | 1 | 2.58E-15 |
| rs1184547 | n/a | n/a | 1 | 2.58E-15 |
| rs12074528 | n/a | n/a | 1 | 2.58E-15 |
| rs7534572 | n/a | n/a | 1 | 2.58E-15 |
| rs7548877 | n/a | n/a | 1 | 2.58E-15 |
| rs4915840 | n/a | n/a | 1 | 2.58E-15 |
| rs2366638 | n/a | n/a | 1 | 2.59E-15 |
| rs746735 | n/a | n/a | 1 | 2.6E-15 |
| rs12029068 | n/a | n/a | 1 | 2.62E-15 |
| rs1781195 | n/a | n/a | 1 | 2.63E-15 |
| rs1168044 | n/a | n/a | 1 | 2.65E-15 |
| rs12062275 | n/a | n/a | 1 | 2.67E-15 |
| rs1168097 | n/a | n/a | 1 | 2.68E-15 |
| rs1168098 | n/a | n/a | 1 | 2.68E-15 |
| rs28641195 | n/a | n/a | 1 | 2.68E-15 |
| rs11207970 | n/a | n/a | 1 | 2.69E-15 |
| rs10789114 | n/a | n/a | 1 | 3.66E-15 |
| rs6587976 | n/a | n/a | 1 | 3.71E-15 |
| rs10889354 | n/a | n/a | 1 | 4.11E-15 |
| rs1690766 | n/a | n/a | 1 | 4.91E-15 |
| rs1168015 | n/a | n/a | 1 | 5.23E-15 |
| rs2479464 | n/a | n/a | 1 | 5.74E-15 |
| rs638714 | n/a | n/a | 1 | 1.43E-14 |
| rs9436221 | n/a | n/a | 1 | 1.66E-14 |
| rs9436222 | n/a | n/a | 1 | 1.66E-14 |
| rs10889331 | n/a | n/a | 1 | 4.08E-14 |
| rs10889330 | n/a | n/a | 1 | 4.3E-14 |
| rs2131925 | 8.84E-43 | 1.64E-17 | 1 | 2.14E-15 |
| rs10889347 | 1.23E-42 | 2.24E-17 | 1 | 2.42E-15 |
| rs4329540 | 1.28E-42 | 2.71E-17 | 1 | 2.14E-15 |
| rs10889350 | 2.43E-42 | 1.73E-17 | 1 | 2.49E-15 |
| rs1748195 | 2.43E-42 | 3.17E-17 | 1 | 2.11E-15 |
| rs10159255 | 2.49E-42 | 1.54E-17 | 1 | 3.66E-15 |
| rs11207992 | 2.51E-42 | 1.45E-17 | 1 | 3.66E-15 |
| rs1748197 | 2.75E-42 | 1.46E-17 | 1 | 2.28E-15 |
| rs11207990 | 3.01E-42 | 1.49E-17 | 1 | 2.14E-15 |
| rs1748200 | 3.06E-42 | 1.46E-17 | 1 | 2.11E-15 |
| rs7539035 | 3.16E-42 | 1.32E-17 | 1 | 2.6E-15 |
| rs10889349 | 3.47E-42 | 1.95E-17 | 1 | 2.43E-15 |
| rs6587980 | 3.72E-42 | 1.51E-17 | 1 | 2.5E-15 |
| rs6678483 | 3.83E-42 | 1.26E-17 | 1 | 2.42E-15 |
| rs11207997 | 4.77E-42 | 1.43E-17 | 1 | 2.31E-15 |
| rs1168042 | 9.03E-42 | 9.52E-17 | 1 | 2.47E-15 |
| rs6675401 | 9.06E-42 | 2.08E-17 | 1 | 2.42E-15 |
| rs1627591 | 1.38E-41 | 4.99E-17 | 1 | 2.63E-15 |
| rs1168036 | 1.84E-41 | 1.24E-16 | 1 | 7.29E-15 |
| rs11208000 | 7.06E-41 | 4.64E-18 | 1 | 2.62E-15 |
| rs1168089 | 8.54E-41 | 2.74E-17 | 1 | 2.6E-15 |
| rs634341 | 9.72E-41 | 4.32E-17 | 1 | 1.9E-15 |
| rs630144 | 9.88E-41 | 4.89E-17 | 1 | 2.52E-15 |
| rs1168018 | 9.9E-41 | 6.67E-18 | 1 | 2.58E-15 |
| rs583609 | 1.16E-40 | 5.41E-17 | 1 | 2.05E-15 |
| rs1168013 | 1.57E-40 | 3.95E-18 | 1 | 2.62E-15 |
| rs642845 | 1.81E-40 | 2.64E-17 | 1 | 2.02E-15 |
| rs641540 | 2.21E-40 | 3.49E-17 | 1 | 2.1E-15 |
| rs10158897 | 2.66E-40 | 3.72E-17 | 1 | 2.99E-15 |
| rs597078 | 2.98E-40 | 8.85E-17 | 1 | 1.97E-15 |
| rs638305 | 3.94E-40 | 2.71E-17 | 1 | 2.23E-15 |
| rs637723 | 7.33E-40 | 4.84E-17 | 1 | 2.25E-15 |
| rs1168132 | 8.71E-40 | 2.07E-16 | 1 | 2.42E-15 |
| rs1168086 | 2.59E-39 | 3.39E-17 | 1 | 2.6E-15 |
| rs10493322 | 6.97E-39 | 2.97E-17 | 1 | 2.89E-15 |
| rs659656 | 7.67E-39 | 1.04E-16 | 1 | 2.11E-15 |
| rs1168085 | 6.89E-38 | 5.17E-17 | 1 | 2.6E-15 |
| rs626787 | 1.43E-35 | 1.62E-14 | 1 | 2.43E-15 |
| rs636497 | 1.25E-16 | n/a | 1 | 1.9E-15 |
| rs1168009 | 3E-16 | n/a | 1 | 4.18E-14 |
| rs656297 | 2.82E-15 | n/a | 1 | 2.12E-15 |
| rs597076 | n/a | n/a | 0.965 | 2.07E-15 |
| rs1472257 | n/a | n/a | 0.965 | 2.11E-15 |
| rs6696502 | n/a | n/a | 0.965 | 2.14E-15 |
| rs1168046 | n/a | n/a | 0.965 | 2.49E-15 |
| rs9436223 | n/a | n/a | 0.965 | 2.52E-15 |
| rs11208001 | n/a | n/a | 0.965 | 2.56E-15 |
| rs7520810 | n/a | n/a | 0.965 | 2.57E-15 |
| rs10889340 | n/a | n/a | 0.965 | 2.58E-15 |
| rs11207985 | n/a | n/a | 0.965 | 2.58E-15 |
| rs1179765 | n/a | n/a | 0.965 | 2.58E-15 |
| rs11207976 | n/a | n/a | 0.965 | 2.58E-15 |
| rs1168027 | n/a | n/a | 0.965 | 3.06E-15 |
| rs10789116 | n/a | n/a | 0.965 | 3.66E-15 |
| rs12116574 | n/a | n/a | 0.965 | 3.66E-15 |
| rs1305521 | n/a | n/a | 0.965 | 3.89E-15 |
| rs10789113 | n/a | n/a | 0.965 | 3.9E-15 |
| rs10889338 | n/a | n/a | 0.965 | 3.92E-15 |
| rs11207977 | n/a | n/a | 0.965 | 4.03E-15 |
| rs11207982 | n/a | n/a | 0.965 | 4.09E-15 |
| rs10889348 | n/a | n/a | 0.965 | 4.1E-15 |
| rs11207980 | n/a | n/a | 0.965 | 4.25E-15 |
| rs4495740 | n/a | n/a | 0.965 | 4.49E-15 |
| rs12047226 | n/a | n/a | 0.965 | 9.08E-15 |
| rs1690761 | n/a | n/a | 0.965 | 9.2E-15 |
| rs10789112 | n/a | n/a | 0.965 | 1.5E-14 |
| rs1305520 | n/a | n/a | 0.965 | 2.71E-14 |
| rs35529421 | n/a | n/a | 0.965 | 1.34E-13 |
| rs1168099 | 1.47E-42 | 1.5E-17 | 0.965 | 2.68E-15 |
| rs4587594 | 2.12E-42 | 1.02E-17 | 0.965 | 4.49E-15 |
| rs1781221 | 3.98E-42 | 1.83E-17 | 0.965 | n/a |
| rs1748201 | 4.4E-42 | 1.66E-17 | 0.965 | 2.11E-15 |
| rs10889353 | 6.01E-42 | 1.8E-17 | 0.965 | 4.49E-15 |
| rs11577840 | 6.38E-42 | 3.23E-17 | 0.965 | 3.38E-15 |
| rs1168031 | 6.59E-42 | 5.44E-17 | 0.965 | 5.01E-14 |
| rs1781212 | 7.91E-42 | 4.28E-17 | 0.965 | 2.73E-15 |
| rs1168032 | 8.82E-42 | 5.49E-17 | 0.965 | 8.28E-14 |
| rs1168124 | 9.01E-42 | 2.83E-17 | 0.965 | 7.5E-15 |
| rs11207995 | 9.97E-42 | 2.16E-17 | 0.965 | 3.66E-15 |
| rs1168030 | 1.11E-41 | 4.88E-17 | 0.965 | 5.01E-14 |
| rs10889337 | 1.68E-41 | 4.8E-17 | 0.965 | 4.27E-14 |
| rs1002687 | 2.33E-41 | 8.81E-17 | 0.965 | 9.22E-14 |
| rs10889333 | 3.18E-41 | 6.01E-17 | 0.965 | 1.62E-14 |
| rs1979722 | 4.45E-41 | 6.25E-17 | 0.965 | 4.72E-15 |
| rs10889334 | 4.98E-41 | 4.38E-17 | 0.965 | 1.62E-14 |
| rs1167998 | 5.02E-41 | 8.62E-17 | 0.965 | 3.23E-14 |
| rs11207974 | 5.3E-41 | 3.11E-17 | 0.965 | 4E-15 |
| rs11208004 | 6.78E-41 | 2.08E-17 | 0.965 | 2.42E-14 |
| rs1168022 | 8.28E-41 | 7.4E-17 | 0.965 | 3.93E-15 |
| rs597470 | 9.82E-41 | 6.19E-17 | 0.965 | 1.96E-15 |
| rs1183260 | 1.16E-40 | 9.29E-17 | 0.965 | 3.62E-15 |
| rs783291 | 1.22E-40 | 3.42E-17 | 0.965 | 2.09E-15 |
| rs1168023 | 1.65E-40 | 1.43E-16 | 0.965 | 1.9E-15 |
| rs10889352 | 1.9E-40 | 3.13E-18 | 0.965 | 4.35E-15 |
| rs10789119 | 2.59E-40 | 3.59E-18 | 0.965 | 4.35E-15 |
| rs1570694 | 2.82E-40 | 3.33E-18 | 0.965 | 4.38E-15 |
| rs3850634 | 3.11E-40 | 2.63E-18 | 0.965 | 3.66E-15 |
| rs10789118 | 3.19E-40 | 2.89E-18 | 0.965 | 4.35E-15 |
| rs11485618 | 4.16E-40 | 2.7E-18 | 0.965 | 4.35E-15 |
| rs11207981 | 9.43E-40 | 4.89E-17 | 0.965 | 2.58E-15 |
| rs1184865 | 1.64E-39 | 1.19E-17 | 0.965 | 4.79E-14 |
| rs10889332 | 1.91E-39 | 6.7E-15 | 0.965 | 7.43E-15 |
| rs631106 | 3.52E-39 | 3.96E-17 | 0.965 | 2.4E-15 |
| rs3913007 | 9.4E-17 | n/a | 0.965 | 2.55E-15 |
| rs1168040 | 2.28E-16 | n/a | 0.965 | 1.13E-11 |
| rs1168017 | n/a | n/a | 0.932 | 2.58E-15 |
| rs1168102 | n/a | n/a | 0.932 | 2.68E-15 |
| rs10889357 | n/a | n/a | 0.932 | 6.52E-15 |
| rs6666816 | n/a | n/a | 0.932 | 1.19E-14 |
| rs10889360 | n/a | n/a | 0.932 | 1.66E-14 |
| rs9787156 | n/a | n/a | 0.932 | 1.67E-14 |
| rs1168029 | n/a | n/a | 0.932 | 2.94E-14 |
| rs1168002 | n/a | n/a | 0.932 | 3.43E-14 |
| rs1007205 | 5.25E-41 | 4.76E-17 | 0.932 | 1.47E-13 |
| rs1168114 | 1.09E-38 | 7.75E-17 | 0.932 | 5.72E-15 |
| rs1168113 | 5.41E-38 | 1.51E-16 | 0.932 | 5.58E-15 |
| rs880694 | 5.1E-37 | 1.52E-16 | 0.932 | 1.67E-14 |
| rs11208007 | 1.34E-36 | 2.26E-16 | 0.932 | 1.74E-14 |
| rs6682423 | 2.38E-36 | 1.69E-16 | 0.932 | 1.19E-14 |
| rs4409689 | 3.05E-36 | 1.94E-16 | 0.932 | 1.67E-14 |
| rs1168107 | 8.95E-36 | 1.86E-16 | 0.932 | 4.76E-15 |
| rs9787151 | 3.45E-35 | 2.71E-16 | 0.932 | 1.67E-14 |
| rs9436661 | n/a | n/a | 0.931 | 2.59E-15 |
| rs12038768 | n/a | n/a | 0.931 | 3.66E-15 |
| rs34465969 | n/a | n/a | 0.931 | 4.47E-14 |
| rs2029763 | 4.57E-42 | 2.18E-17 | 0.931 | 4.09E-15 |
| rs995000 | 4.74E-42 | 5.18E-17 | 0.931 | 4.4E-15 |
| rs636523 | 9.72E-41 | 5.16E-17 | 0.931 | 2.42E-15 |
| rs12042319 | 2.31E-39 | 3.47E-18 | 0.931 | 3.66E-15 |
| rs11207996 | n/a | n/a | 0.93 | 3.66E-15 |
| rs10889336 | n/a | n/a | 0.93 | n/a |
| rs10889335 | 1.16E-40 | 1.48E-17 | 0.93 | 5.18E-13 |
| rs998403 | 6.99E-40 | 2.13E-17 | 0.93 | 3.61E-15 |
| rs4350231 | 7.53E-39 | 2.98E-17 | 0.93 | 3.01E-14 |
| rs12403207 | n/a | n/a | 0.901 | 2.72E-15 |
| rs1168028 | n/a | n/a | 0.901 | 4.99E-14 |
| rs1168034 | n/a | n/a | 0.901 | 8.7E-14 |
| rs12090886 | 7.34E-42 | 5.35E-17 | 0.901 | 4.08E-14 |
| rs1168026 | 2.01E-40 | 2.18E-17 | 0.901 | 4.76E-14 |
| rs1168010 | 1.28E-16 | n/a | 0.901 | 4.18E-14 |
| rs10889356 | n/a | n/a | 0.897 | 7.55E-15 |
| rs1168045 | 7.96E-42 | 3.42E-17 | 0.897 | 2.49E-15 |
| rs12136083 | 1.62E-37 | 1.9E-16 | 0.897 | 7.24E-15 |
| rs7518497 | 1.3E-35 | 1.7E-16 | 0.897 | 1.74E-14 |
| rs12023489 | n/a | n/a | 0.896 | 2.1E-15 |
| rs7555577 | 1.71E-40 | 1.94E-17 | 0.896 | 7.47E-15 |
| rs78390579 | n/a | n/a | 0.87 | 4.65E-14 |
| rs9436224 | n/a | n/a | 0.862 | n/a |
| rs993013 | n/a | n/a | 0.841 | 2.32E-15 |
| rs6686331 | n/a | n/a | 0.841 | 1.66E-14 |
| rs35194512 | n/a | n/a | 0.835 | n/a |

253 SNPs associated with TG, LDL-C from Teslovich et al, SNPs r^2^ > 0.8 to the TG lead SNP, rs2131925 and liver eQTL SNPs at *ANGPTL3* locus

Supplementary Figure 1


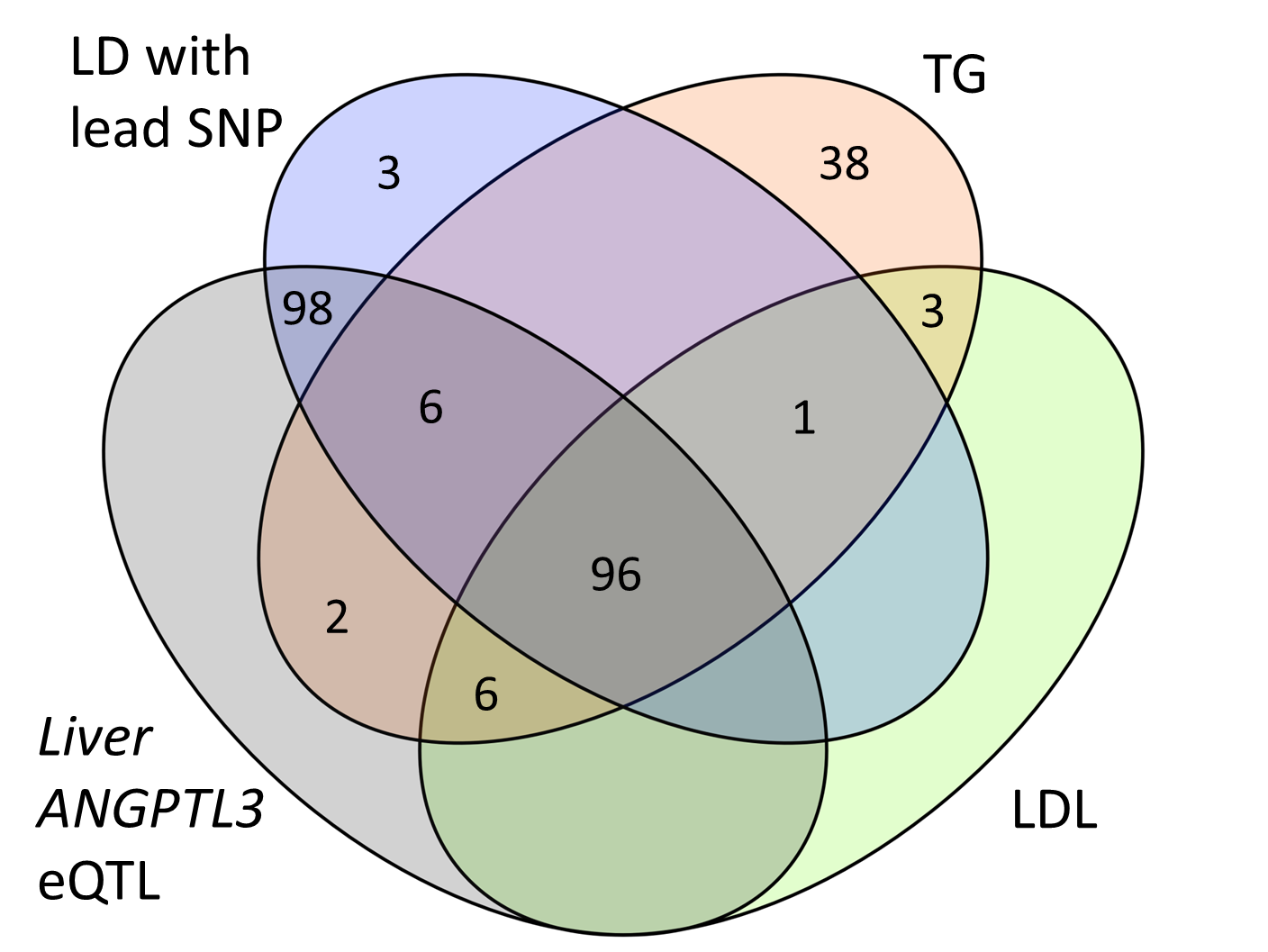


Supplementary Figure 1.

Association of *ANGPTL3* SNPs with TG, LDL-C, liver eQTLs for *ANGPTL3* and SNPs r^2^ > 0.8 with lead TG SNP (rs2131925).

Supplementary Figure 2


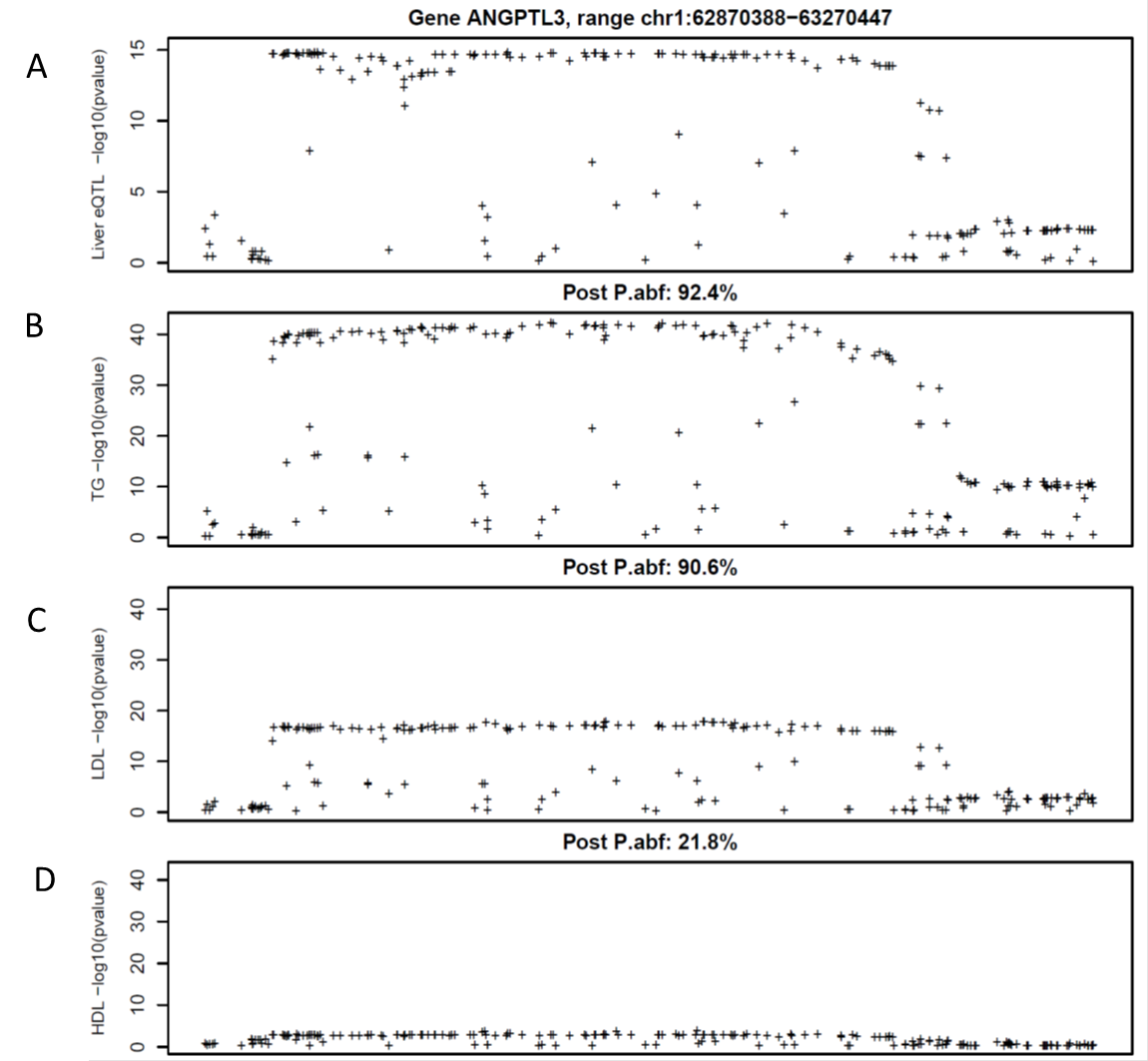


Supplementary Figure 2

Liver eQTL and multiple lipid traits for *ANGPTL3*. The x-axis shows the physical position on the chromosome (Mb). A) y-axis represents association with *ANGPTL3* expression in liver -log^10^(*p*). Each cross represents one SNP (imputed or directly typed). The *p*-values were calculated by fitting a generalized linear model with *ANGPTL3* expression as dependent variable and SNP genotypes as independent variable. B) y-axis uses p-values from a published meta-analysis of triglycerides (TG) levels in >100,000 individuals [[1](#_ENREF_1)]. The posterior probability (Post P.abf) that the TG biomarker co-localises with *ANGPTL3* liver expression is shown on top of the graph. C) Association using the published meta-analysis p-values for LDL and the probability of LDL-C co-localising with the liver eQTL. D) Computations as for plot (C) using published meta-analysis *p*-values for HDL-C. The posterior probability of having a shared variant is high for TG and LDL-C and low for HDL-C, suggesting that variants that mediate the expression of *ANGPTL3* also mediate the effect of TG and LDL-C biomarkers, whereas these do not affect HDL-C levels.

Supplementary Figure 3


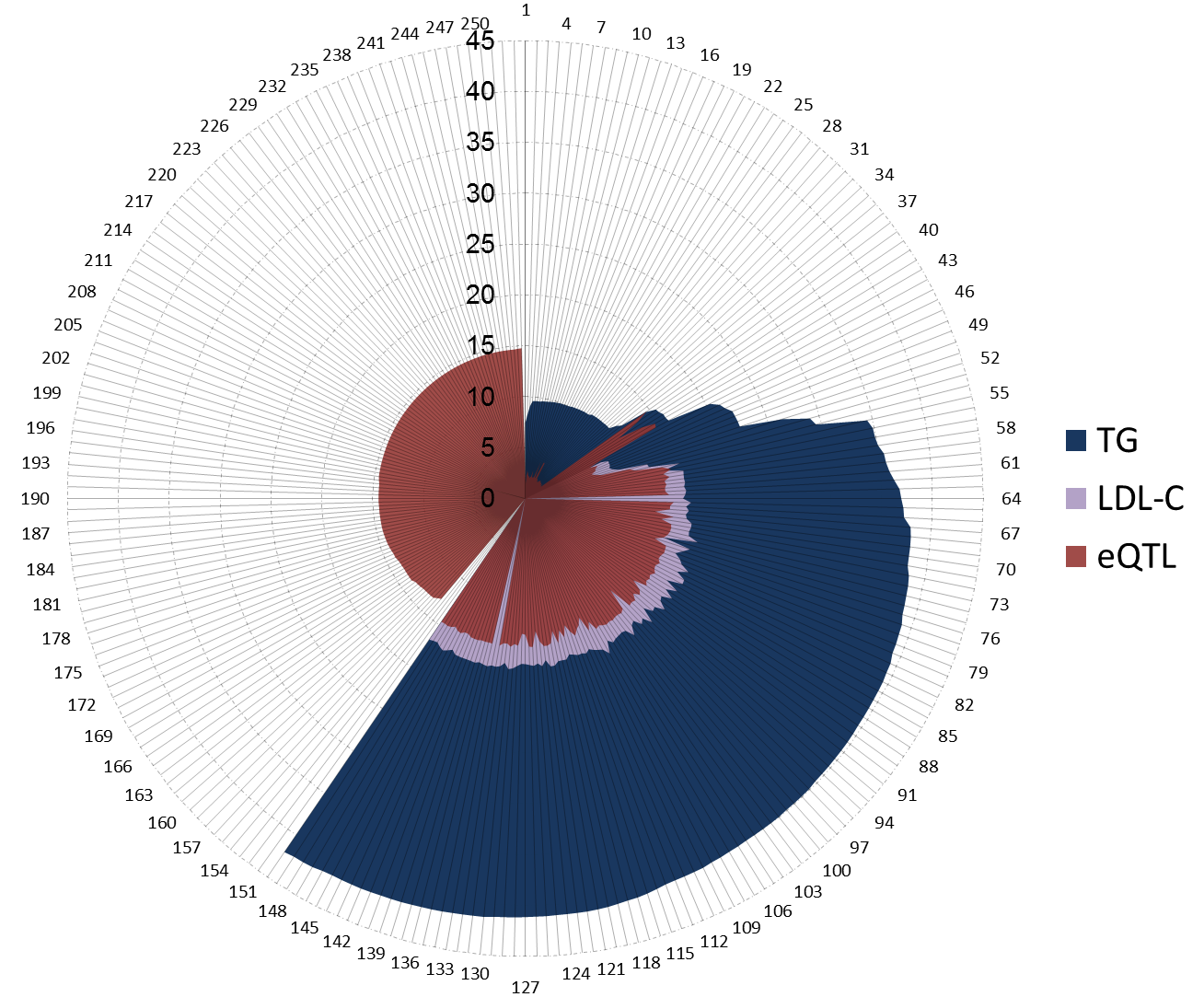


Supplementary Figure 3.

Association of 253 SNPs at the ANGPTL3 locus with TG levels, LDL-C levels and liver *ANGPTL3* eQTLs. SNPs with no reported association to lipid levels on the plot represent those in strong LD with the TG GWAS lead SNP (rs2131925). The y-axis represents –log^10^p for the associations indicated. All LDL-C SNPs are contained within the TG-associated SNPs. TG and LDL-C association significance correlates with *ANGPTL3* eQTLs.

Supplementary Figure 4


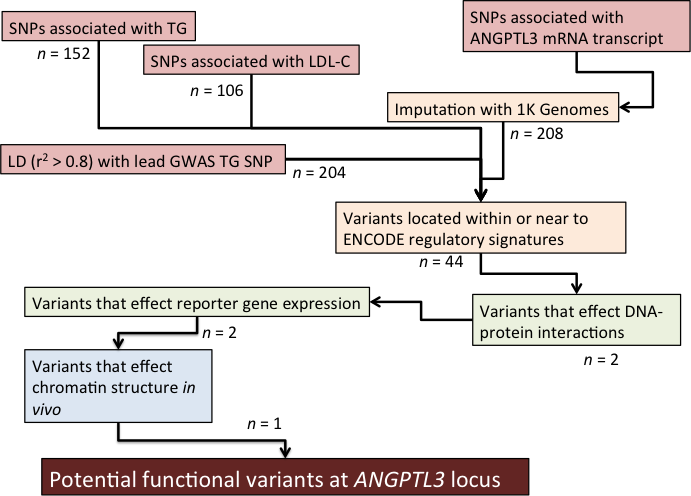


Supplementary Figure 4

Flow of bioinformatics and laboratory methodology used to identify functional variation at the *ANGPTL3* locus

Supplementary Figure 5


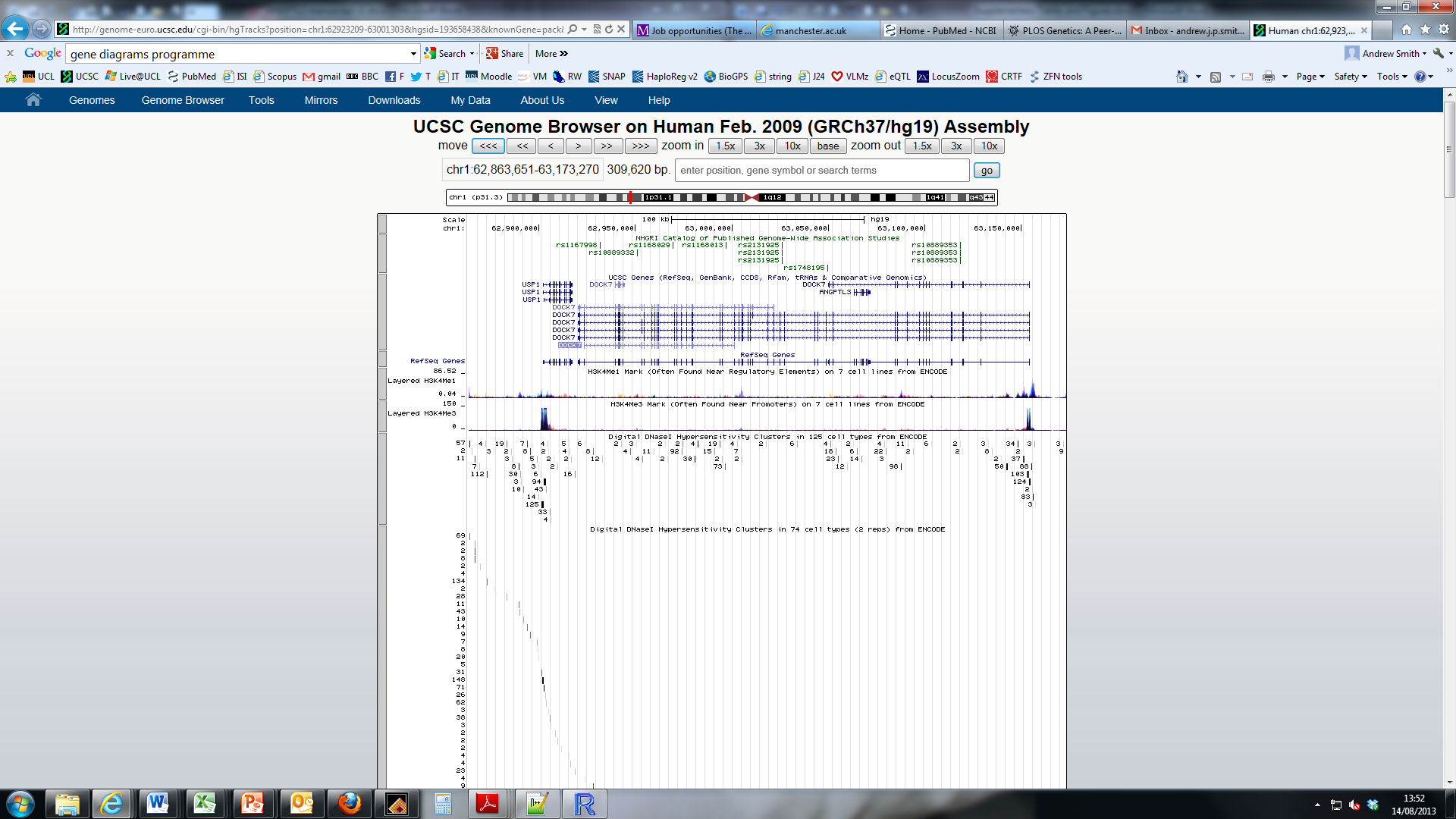


rs10889352

Supplementary Figure 5

Location of putative functional SNP relative to *ANGPTL3* and *DOCK7* transcripts. Variant lies within *DOCK7*, but outside of *ANGPTL3*, suggesting long-range regulatory roles in gene expression.

Supplementary Figure 6


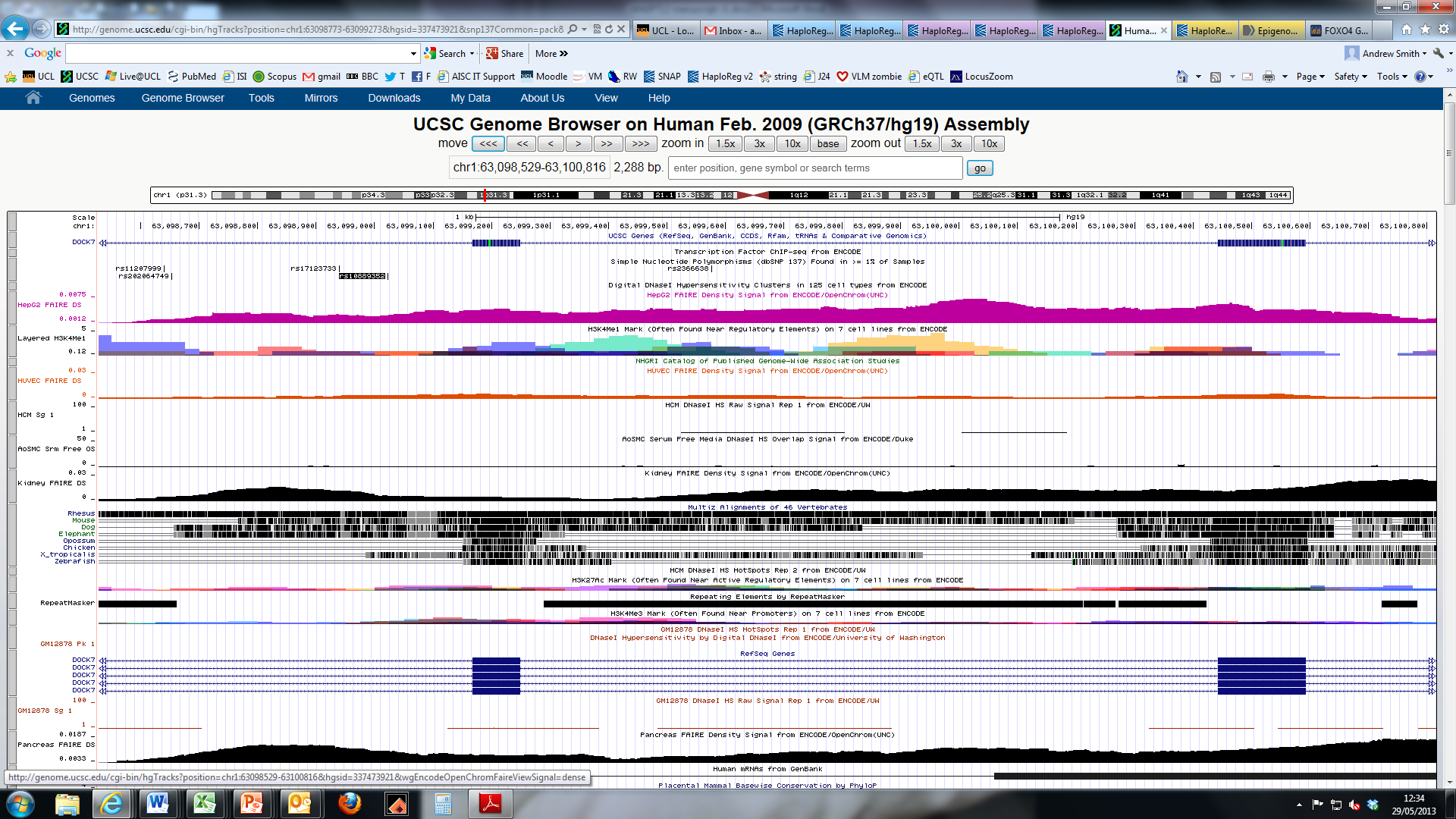


rs10889352

rs6690733


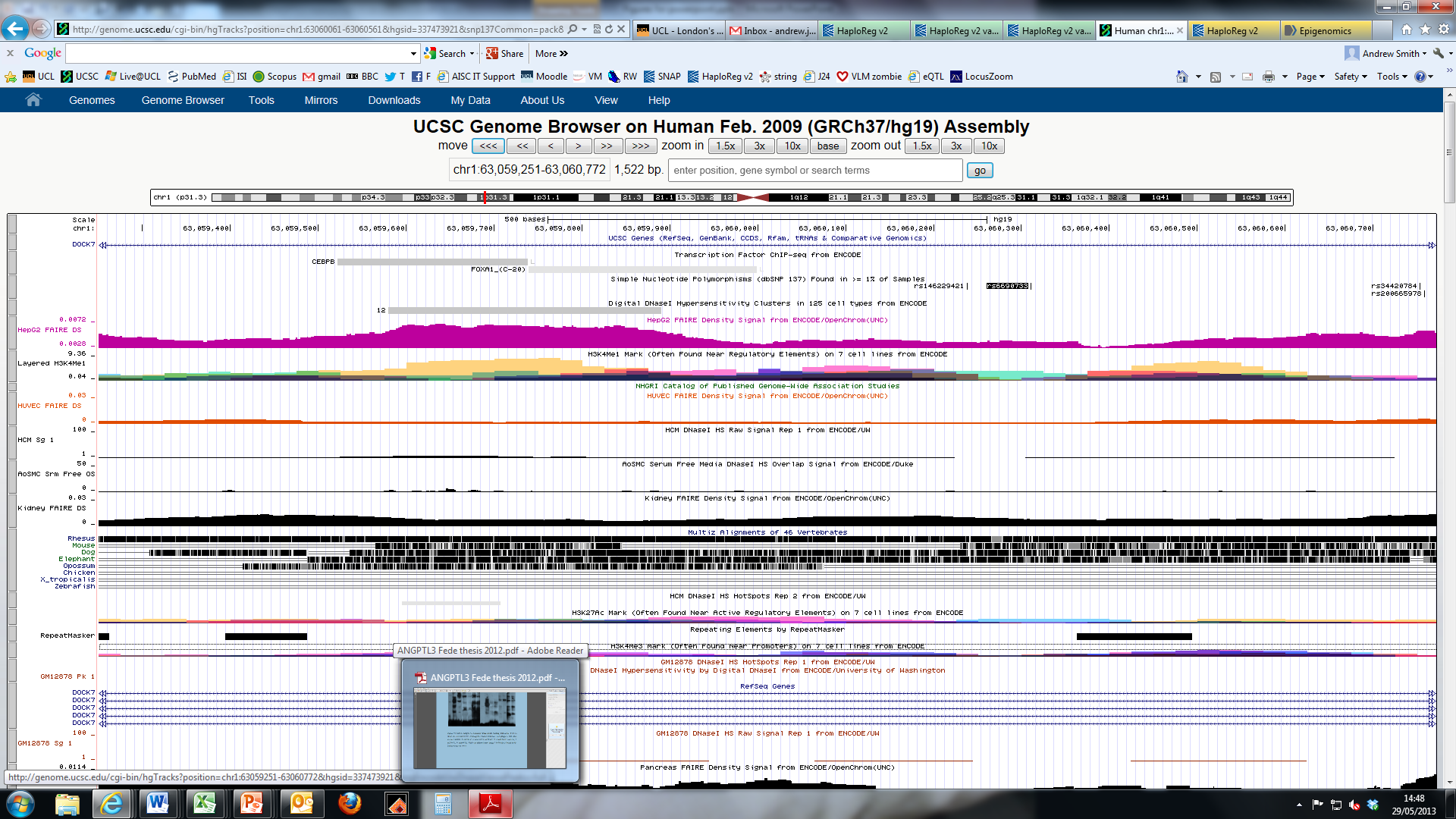


Supplementary Figure 6

ENCODE annotations surrounding the two variants shown to alter DNA-protein interactions *in vitro*. The SNP is highlighted in black, DNaseI sites and transcription factor binding sites are denoted by grey boxes. Open chromatin peaks from the HepG2 liver cell line is shown in magenta.
